# Substrate specificity and safener inducibility of the plant UDP‐glucose‐dependent family 1 glycosyltransferase super‐family

**DOI:** 10.1111/pbi.12775

**Published:** 2017-07-17

**Authors:** Melissa Brazier‐Hicks, Markus Gershater, David Dixon, Robert Edwards

**Affiliations:** ^1^ School of Agriculture, Food and Rural Development Newcastle University Newcastle upon Tyne UK; ^2^ Synthace Ltd London UK; ^3^ GlaxoSmithKline Stevenage UK

**Keywords:** Arabidopsis, rice, xenobiotics, glucosyltransferase, cytochrome P450, glutathione transferase

## Abstract

Plants contain large numbers of family 1 UDP‐glucose‐dependent glycosyltransferases (UGTs), including members that conjugate xenobiotics. Arabidopsis contains 107 UGT genes with 99 family members successfully expressed as glutathione transferase (GST)‐fusion proteins in *E. coli*. A high‐throughput catalytic screen was developed based on quantification of the fusion by measuring GST activity. UGT activity using UDP‐glucose as donor was then determined using 11 synthetic acceptors bearing hydroxyl, amino and thiol groups that had been shown to undergo conjugation in plant extracts. In total, 44 UGTs, largely members of the D and E groups, were active towards xenobiotics, glucosylating phenol and thiol acceptors. In contrast, *N‐*glucosyltransferase (NGT) activity was almost exclusively restricted to a single enzyme, UGT72B1. Using DNA microarrays, the induction of UGT transcripts following treatment with the herbicide safener fenclorim was compared in Arabidopsis and rice. D and L group members were the most safener‐inducible UGTs in both species. The respective Arabidopsis enzymes showed low conjugating activity towards xenobiotics. Using Genevestigator, a small group of safened D and L UGTs were consistently induced in response to biotic and abiotic stress suggestive of protective activities beyond xenobiotic detoxification in both species. The induction of other detoxifying gene families following treatment with fenclorim, namely cytochromes P450 and glutathione transferases, further confirmed the selective enhancement of related subfamily members in the two species giving new insight into the safening response in cereals, where herbicide tolerance is enhanced compared with dicots, which are unresponsive to these treatments.

## Introduction

Plants have evolved a comprehensive chemical detoxification system we have termed the xenome (Edwards *et al*., 2005), that has many parallels with the drug metabolizing systems of animals (Bártíková *et al*., 2015). In common with animals, the xenome of plants has the capacity to metabolize a diverse range of foreign compounds (xenobiotics), including microbial phytotoxins, allelochemicals, pollutants, pesticides and pollutants (Bártíková *et al*., 2015; Cole and Edwards, 2000). However, plants are also able to endogenously produce toxic secondary metabolites with the control of their bioactivity also requiring the intervention of the xenome (Cole and Edwards, 2000). This relationship between the role of the xenome in endogenous secondary and xenobiotic metabolism is poorly understood. The plant xenome is composed of large families such as the glutathione transferases (GSTs), cytochrome P450s (CYPs) and family 1 glycosyltransferases (UGTs) that catalyse the detoxification of xenobiotics, whose coordinated response is underpinned by a network of regulatory proteins (Dixon *et al*., 2009; Nelson *et al*., 2004; Ross *et al*., 2001; Behringer *et al*., 2011). The resulting detoxification products are then commonly transported into the vacuole for further processing and long‐term sequestration (Cole and Edwards, 2000). In addition, extracellular export (Brazier‐Hicks and Edwards, 2005) and incorporation into insoluble bound residues such as lignin (Brazier‐Hicks *et al*., 2007a) are also used by plants to detoxify xenobiotics. As such, the plant xenome plays a key role in removing chemical residues from the environment (Widdup *et al*., 2015) and in defining the bioavailability and hence efficacy of crop protection agents, notably herbicides (Cole and Edwards, 2000). In the latter case, the manipulation of the xenome is of great agricultural interest, with its induction in cereals by safeners enhancing herbicide tolerance and hence selectivity, in the crop (Skipsey *et al*., 2011). Intriguingly, while safeners are known to induce xenome genes in both monocotyledonous and dicotyledenous (dicot) plants (Edwards *et al*., 2005), enhanced herbicide tolerance is only seen in cereal crops (Skipsey *et al*., 2011). This strongly suggests that the ‘xenobiotic response’ associated with safeners is fundamentally different in cereals as compared with dicots.

By applying functional genomics approaches, it is now possible to unravel the complex function and regulation of xenome components such as CYPs (Nelson *et al*., 2004), GSTs (Dixon *et al*., 2009) and UGTs (Caputi *et al*., 2012). We are particularly interested in identifying the critical members of these gene families responsible for pesticide detoxification, their relationship to endogenous secondary metabolism and their differential regulation and functional link to herbicide safening. Of these enzymes, the UGTs are an attractive group of xenome components to explore functional and regulatory genomics. Thus, UGTs are well known to have roles in endogenous metabolism (Bowles *et al*., 2006), to be safener inducible (Edwards *et al*., 2005) and amenable to mass expression and screening studies (Brazier‐Hicks *et al*., 2007a,b). The family 1 UGTs involved in small molecule glycosylation are one of the largest of 103 glycosyltransferase families identified in bacteria, fungi, animals and plants (http://www.cazy.org/). UGTs glycosylate a variety of functional groups, including carboxyl and hydroxyl groups to produce *O*‐glycosides, amine and sulphydryl groups to produce *N*‐ and *S*‐glycosides, respectively, and can also catalyse the formation of *C*‐glycosides (Tiwari *et al*., 2016). In plants, UGT sequences can be identified by the presence of the conserved plant secondary product glycosyltransferase (PSPG) motif which is involved in sugar donor binding and located in the C‐terminal region (Bowles *et al*., 2006). The majority of plant UGTs characterized to date use uridine diphosphoglucose (UDPG) as the sugar donor (Osmani *et al*., 2008). Genome sequencing has revealed that UGTs are present throughout the plant kingdom ***.*** While only one UGT was identified in the single‐celled green algae *Chlamydomonas reinhardtii* **,** higher plants such as rice (*Oryza sativa* L.) and sorghum (*Sorghum bicolor* L.) each contain around 180 UGTs, which have evolved into 17 (A‐Q) phylogenetic groups (Caputi *et al*., 2012). The ability of UGTs to conjugate xenobiotics is also universal in plants. When 59 species including algae, mosses, ferns, conifers, and crops were assayed, it was found that *O*‐glucosyltransferase (OGT) activity towards chlorinated phenols was present in all plants tested (Pflugmacher and Sandermann, 1998).

**Figure 1:**
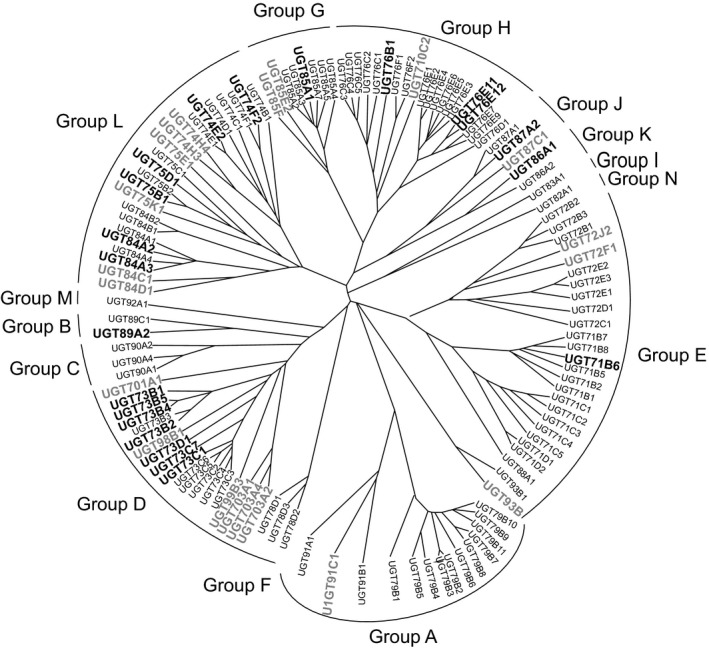
Phylogenetic tree of UGTs forming this study. For reference genes up‐regulated by a 4‐h treatment with 100 μm fenclorim in Arabidopsis, root cultures and the orthologues in rice suspension cultures are shown in bold black and grey type, respectively.

In *Arabidopsis thaliana*, there are 120 UGT genes of which 107 encode apparently functional enzymes (Bowles *et al*., 2006). These can be organized into 14 groups based on sequence similarity and evolutionary relatedness (Figure 1). These Arabidopsis UGTs have been subjected to detailed analysis with respect to their activities towards endogenous secondary metabolites and hormones (Bowles *et al*., 2006). While sugar donor specificities are largely conserved within the groups, the selectivity towards acceptor cosubstrates is less predictable. Based on studies with orthologues in other plant species, some of the smaller groups do have conserved acceptor substrate specificity. For example, group F members selectively conjugate the 3‐hydroxy position of flavonols (Ono *et al*., 2010), while all zeatin‐*O*‐glucosyltransferases discovered so far belong to group O (Caputi *et al*., 2012). In contrast, members of the larger phylogenetic groups from a variety of plant species conjugate a wide range of aglycones (Tiwari *et al*., 2016). With respect to xenobiotic glucosylation in Arabidopsis, six UGTs with known activities towards endogenous phenolic and carboxylic acid bearing substrates also catalysed the *O*‐ether conjugation of 2,4,5‐trichlorophenol (Meßner *et al*., 2003). Subsequent studies further utilized 2,4,5‐trichlorophenol (2,4,5‐TCP) as a reactive *O‐*acceptor to demonstrate that a total of 44 Arabidopsis UGTs, predominantly belonging to the D, E and L groups, were active towards this xenobiotic (Brazier‐Hicks *et al*., 2007a,b). Six members of the D‐group UGTs were also found to have a mixture of OGT and *C*‐glucosyltransferase (CGT) activity towards degradation products of trinitrotoluene (Gandia‐Herrero *et al*., 2008).

We now report on a systematic study to define a plant UGT xenome. Using enzyme activity screens of group members from Arabidopsis, we have tested the respective UGT super‐family for their ability to conjugate a range of xenobiotic acceptor substrates. Using the safener fenclorim, we have then used microarray technology to compare changes in the transcriptome of UGT family members in this model dicot plant with that determined in rice. Fenclorim was selected as it is used commercially to protect rice from injury by chloroacetanilide herbicides and was an optimal inducer of GSTs in Arabidopsis cultures when tested with other safener chemistries (Skipsey *et al*., 2011). Extending this analysis to the CYP and GST gene families, we have then compared the induction of the wider xenome in safened rice as compared with Arabidopsis, where fenclorim treatment does not enhance herbicide tolerance.

## Results

### The range of xenobiotics glucosylated by Arabidopsis

**Figure 2:**
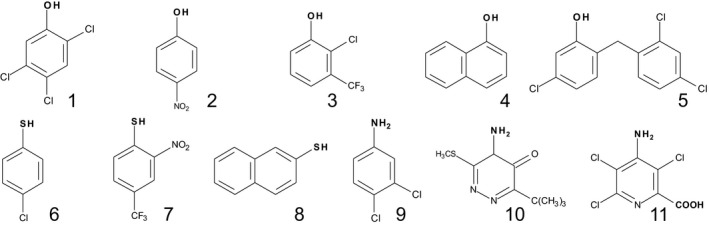
Glucosyltransferase activities determined in Arabidopsis plan extracts. (a) Structures of the eleven glucose xenobiotic acceptors identified as substrates and (b) activities determined in root and foliar tissue. Potential groups serving as sites for glucosylation are shown in bold. OGT substrates; 2,4,5‐trichlorophenol [1], 4‐nitrophenol [2], 2‐chloro‐4‐trifluoromethylphenol [3], 1‐naphthol [4], triclosan [5]. SGT substrates; 4‐chlorothiophenol [6], 2‐nitro(4‐trifluoromethyl)‐benzenethiol [7], 2‐naphthalenethiol [8]. NGT substrates; 3,4‐dichloroaniline [9], metribuzin, [10], picloram [11].

To define the diversity of xenobiotic conjugating UGT activity in plants, crude protein extracts from the foliage and roots of Arabidopsis were assayed for their glucosylating activity towards 30 xenobiotic acceptors (Table S1). The sugar acceptors were selected based on their use as crop protection agents (Cole and Edwards, 2000), description as priority pollutants (Pflugmacher and Sandermann, 1998) or as pharmaceuticals that undergo glycosylation in animals (Tripathi *et al*., 2013). The compounds contained at least one functional group (−COOH, −OH, −NH_2,_ −SH) that could undergo carboxy ester, *O*‐, *N*‐ or *S*‐ conjugation, respectively. Based on this screen, 11 of the compounds tested were found to undergo measurable glucosylation when incubated with the Arabidopsis plant extracts and were selected for further study. UGT activity was not observed towards any compound bearing only a carboxyl acceptor. The structures and activities determined in the two tissue types with 11 substrates are shown in Figure 2. On the basis of specific activity per unit protein, conjugation was greatest in the roots. In the case of the herbicides metribuzin and picloram, these compounds were only glucosylated at low rates in root cultures. The most actively conjugated substrates in both roots and shoots, (Figure 2b), were the *O‐*acceptor 2,4,5‐TCP and the *N*‐acceptor 3,4‐dichloroaniline (3,4‐DCA). These two substrates were selected to explore the xenobiotic‐ conjugating activity in a wider range of plant tissues/organs (Table S2). Greatest activities towards both substrates were determined in de‐differentiated cell cultures, foliage and flower tissue, with specific NGT activities being greater than the OGT activities in all the Arabidopsis plant parts sampled.

### Development of a high‐throughput assay for screening recombinant UGTs

In previous studies, 107 UGTs had been cloned from Arabidopsis and expressed as the respective recombinant glutathione transferase (GST)‐fusion proteins in *E. coli* (Ross *et al*., 2001). Conventionally, these fusion proteins are purified by affinity chromatography using the GST‐tag prior to assay. To facilitate the rapid screening of multiple xenobiotic substrates, an alternative method was developed. As the GST protein tag can catalyse the glutathione‐conjugation of the model substrate 1‐chloro‐2,4‐dinitrobenzene (CDNB), it was reasoned that determining its activity in crude recombinant protein extracts could be used to accurately quantify the amount of fusion protein present. Firstly, to determine whether the two enzymes comprising the fusion interfered with each other, the GST was purified by affinity chromatography both as the native protein and cojoined with the enzyme UGT72B1. This UGT was selected as it is both a highly active *N‐*glucosyltransferase (NGT) and OGT (Brazier‐Hicks *et al*., 2007a,b). The pure proteins were then assayed for GST activity towards CDNB. The singly expressed GST and the respective enzyme fused to the UGT had very similar turnover numbers of 1.60 s^−1^ and 1.88 s^−1^, respectively. A native version of UGT72B1 was then purified and compared with GST‐UGT72B1 for glucosyltransferase activity towards 3,4‐DCA. The respective turnover numbers were 0.63 s^−1^ (native) and 0.66 s^−1^ (fusion). These results demonstrated that the two component polypeptides of the fusion did not interfere significantly with one another's catalytic activities. The GST in crude lysates was assayed by Western blotting, using an anti‐GST‐serum conjugated to horseradish peroxidase. The amount of bound antibody was then quantified using a chemifluorescent based assay. The physical amount of GST fusion present in the lysate was directly correlated with the chemifluorescent signal determined on the blots, with *R* ^2^ = 0.98 (Figure S1A), confirming that the westerns were quantitative for immunogenic protein. This analysis was then extended to the GST‐UGT72B1, GST‐UGT74E1, GST‐UGT72B2 and GST‐UGT76E1 fusion proteins. In each case, GST activity towards CDNB in the fusion was correlated with the chemifluorescent intensity determined by quantitative Western blotting Figure S1B). The results showed a linear relationship, confirming that GST activity towards CDNB provided a quantitative measure of the concentration of GST‐UGT fusion. Importantly, the lysates of untransformed *E. coli* possessed negligible GST activity towards CDNB, confirming that the host cells would not interfere with the quantification of the fusion enzyme. For the comparative purposes of the mass screen, the simple GST‐coupled enzyme assay allowed for the rapid quantification of UGT expression in crude lysates and hence the determination of specific glucosyltransferase activities towards multiple substrates.

### Screen of recombinant Arabidopsis UGTs for activity towards xenobiotics

The 11 acceptor substrates identified from screening the plant extracts were used to systematically screen the different groups of recombinant Arabidopsis GST‐UGT fusions. The panel included five xenobiotics containing hydroxyl groups, three with sulphydryl groups and two with amino groups and one (picloram) with both amino and carboxyl moieties (Figure 2a). Of the 107 Arabidopsis UGT‐GST fusion proteins, five did not express as active soluble proteins in *E. coli* and these were not tested further. Screening of the fusion proteins found demonstrated that 58 UGTs showed no activity towards 2,4,5‐TCP. These included group A members that similarly lacked activity towards all natural acceptors tested to date (Bowles *et al*., 2006). In general, UGT activity towards xenobiotics was widely seen in most of the larger groups, with the exception of the H enzymes were the majority were inactive. The other inactive UGTs belonged to the minor groups F, I, J, K and M which all contain three or fewer members. Of the remaining 44 recombinant fusions, 13 UGTs were active towards all three classes of acceptor (*O*‐, *N*‐ and *S‐*), notably a cluster of E‐group (UGT72B1, UGT72B3 and UGT88A1) enzymes (Table 1). Of the *O*‐glucosyl acceptors, 2‐chloro‐4‐trimethylfluoromethylphenol and triclosan were the preferred substrates after 2,4,5‐TCP (Table 1). Members of groups D and E showed the highest and broadest range of activities towards hydroxylated substrates, especially UGT71C1, UGT71C2, UGT88A1 and UGT72B1 (Table 1). UGT71C1 and UGT71C2 were exclusively active as OGTs and uniquely showed appreciable activity towards 1‐naphthol. Unlike OGT conjugation, NGT activity towards amino groups was largely confined to group E (Table 1), notably to the single enzyme UGT72B1 (Brazier‐Hicks *et al*., 2007a,b). UGT72B1 was also highly active towards the phenolic acceptors, conjugating both 2,4,5‐TCP and 2‐chloro‐trimethylfluoromethylphenol some 20‐fold higher than the most active OGT, UGT71C1. The most adaptable enzymes proved to be UGT72B1 and UGT88A1; both catalysed *O*‐, *S*‐ and *N*‐glucosylation reactions with xenobiotics. In particular, UGT88A1 was unique in being able to *N*‐glucosylate the herbicides metribuzin and picloram. Many of the UGTs catalysing *O*‐conjugation also showed *S*‐glucosyltransferase (SGT) activity. Of the most active SGTs, UGT74F1, UGT74B1 and UGT84B1 from group L showed higher specific activities towards all the thiophenols tested than 2,4,5‐TCP, suggestive of a selectivity for *S‐*acceptors (Table 1). While xenobiotic conjugating activity was widespread in the D and E groups, only one member from each of groups B, C, F and G was active towards the panel (Table 1). The activities of UGTs towards specific acceptor groups on natural product substrates did not correlate with the corresponding conjugation of the xenobiotics. Thus, group L members that catalysed the formation of glucose esters with indole‐3‐acetic acid and benzoates (Lim *et al*., 2002) showed no activity towards the xenobiotic carboxylic acids listed in Table S1. Similarly, UGT76C1 and UGT76C2 that both *N*‐glucosylate cytokinins (Hou *et al*., 2004) were unable to conjugate the *N*‐acceptors 3,4‐DCA, metribuzin and picloram.

**Table 1:**
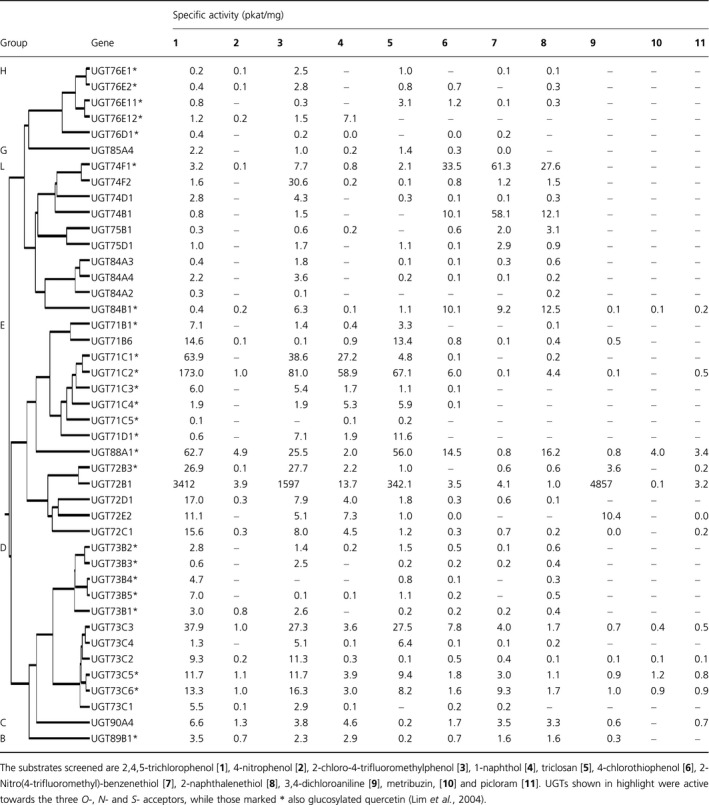
Specific activities of recombinant UGTs towards xenobiotic substrates

### Expression profiling of UGTs from rice and Arabidopsis following treatment with the herbicide safener fenclorim

Microarray transcriptome studies were performed on rice N1 suspension cell cultures treated under identical conditions to that of a previously published study on Arabidopsis cultures (GEO Accession no. GSE 28431) by Skipsey *et al*. (2011). Both sets of cultures were treated with fenclorim (100 μm) and harvested after four and 24 h. Changes in overall gene expression (Table S3) and in expression of xenome genes (Table S4) were then determined over the time course. With respect to the induction of UGTs, in rice of the 151 putative UGTs, 19 were up‐regulated more than threefold (FDR > 0.001) after 4 h (Table S4). Using a similar induction threshold, this compared with 22 UGTs in Arabidopsis. If a higher 10‐fold induction threshold was adopted, six Arabidopsis UGTs were enhanced as compared with just one in rice (Table S4). To compare the phylogenies of UGTs induced by the safener in the two species, the rice genes were assigned identities using the UDP‐glycosyltransferase nomenclature committee database system (Mackenzie, 1997). Two putative rice UGTs on the array, LOC_Os02g36810 and LOC_Os04g46970, had not previously been included in this database. For comparative analysis, a tree was constructed using all 107 Arabidopsis UGT sequences, together with UGT93B1 from *Zea mays* (Martin *et al*., 1999), so as to phylogenetically map the respective inducible rice genes. Using this approach, the two previously undescribed rice UGTs were assigned to subfamilies UGT85F and UGT93B. Overall, the safener‐inducible UGTs in rice and Arabidopsis clustered into 10 previously described phylogenetic groupings (Ross *et al*., 2001; Caputi *et al*., 2012). The combined phylogenetic tree showing the diversity of UGTs induced by the safener treatment is shown in Figure 1. Some clear differences in the responsiveness of the UGTs in the two species to safeners were apparent. Fenclorim treatment uniquely enhanced the group O enzyme UGT93B in rice, an orthologue of a *cis‐*zeatin‐*O*‐glucosyltransferase in *Z. mays*. In contrast, A, B and K group members were only induced in Arabidopsis. Only two of the group E enzymes, the most numerous of all UGTs in both species, were induced by the safener. Instead, the majority of up‐regulated genes in both species belonged to the D and L groups. In group D, it was clear that the families had evolved through different routes in the two species. In Arabidopsis, group D UGTs have evolved from a single family (UGT73). In rice, the D group have arisen from seven families (Caputi *et al*., 2012), of which four contained members up‐regulated by fenclorim treatment. As such, rice contained a more diverse set of safener‐inducible D‐group UGTs than Arabidopsis. By comparison, safener‐inducible L group enzymes were distributed between the three families (UGT74, UGT75 and UGT84), in both species. In the smaller phylogenetic groups, G and J UGTs from both species were induced by fenclorim.

### Comparison of UGT gene induction by safeners and other stress treatments in Arabidopsis and rice

**Figure 3:**
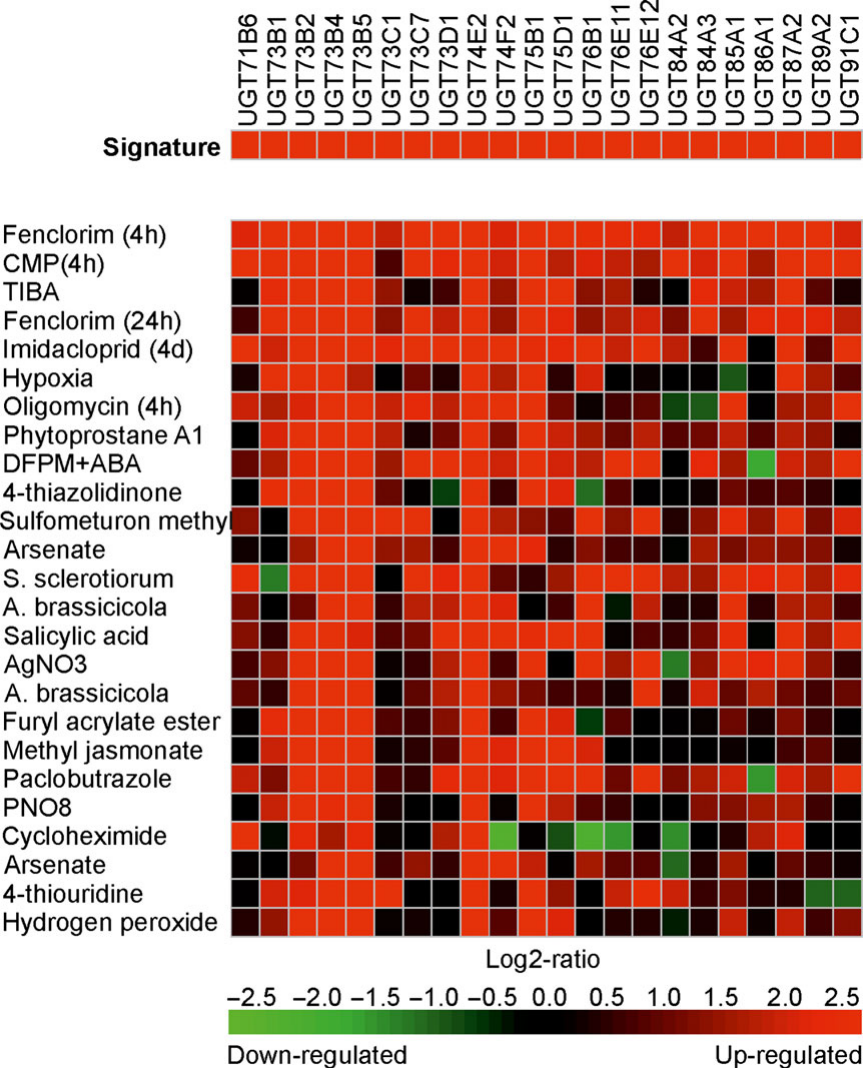
Heat map showing the top 25 conditions as identified by Genevestigator which perturb Arabidopsis UGTs in a similar manner to that determined following treatment with the herbicide safener fenclorim. Red indicates up‐regulation, black no change and green down‐regulation with the colour intensity reflecting the Log2 perturbation.

**Figure 4:**
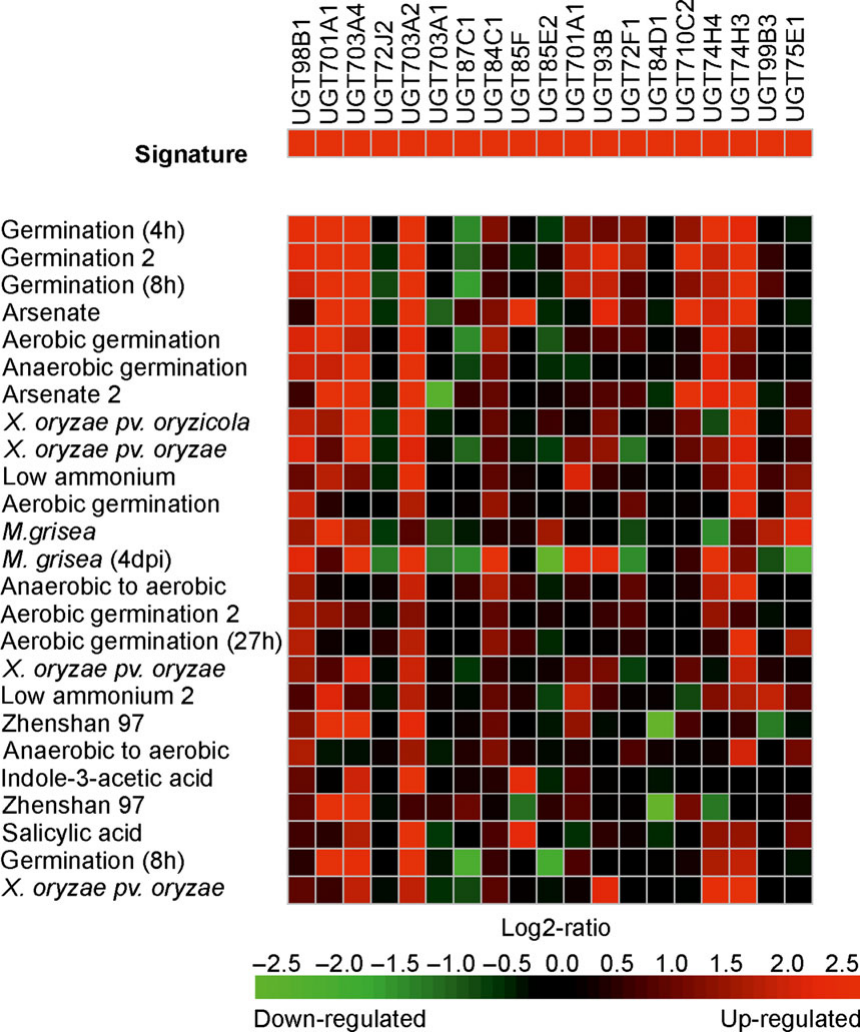
Heat map showing the top 25 conditions as identified by Genevestigator which perturb rice UGTs in a similar manner to that determined following treatment with the herbicide safener fenclorim. Red indicates up‐regulation, black no change and green down‐regulation with the colour intensity reflecting the Log2 perturbation.

It was then of interest to examine the transcriptional regulation of the UGT super‐family in Arabidopsis plants exposed to other chemical treatments. The Genevestigator expression data platform (Hruz *et al*., 2008) is able to interrogate 697 DNA array studies in Arabidopsis representing 5825 perturbations in the transcriptome in response to a diverse range of developmental cues and imposed stresses. In this study, treatments analysed the changes in gene expression resulting from exposure to metabolic inhibitors, plant hormones, natural products and a range of agrochemicals. In rice, the data set was less comprehensive, with only 136 studies covering 1655 experimental conditions. For each species, the UGTs up‐regulated following a 4‐h treatment with fenclorim were used to construct a gene induction signature, which was then used to search for conditions that resulted in similar patterns of perturbation. The 25 signatures most similar to fenclorim treatment were then compared using a ‘heat map’ to compare UGT coregulation in Arabidopsis (Figure 3) and rice (Figure 4).

In Arabidopsis, the D‐group genes UGT73B4 and UGT73B5, together with UGT74E2 from group L, were up‐regulated in all 25 treatments, suggesting these four enzymes are core to the UGT‐stress response in Arabidopsis (Figure 3). Of the comparisons, the greatest similarity with fenclorim treatment was seen following exposure to pesticides, namely the closely related chemical derivative 4‐chloro‐6‐methyl‐2‐phenylpyrimidine (CMP), the insecticide imadacloprid, the sulphonylurea herbicide sulfometuron methyl and the triazole fungicide paclobutrazole. Of the biotic treatments, the most similar induction of UGTs was seen following Infection with the plant fungal pathogens *Sclerotinia sclerotiorum* and *Alternaria brassicicola* and exposure to phytoprostane A1. The response to the phytoprostane oxylipin was of interest, as these fatty acid signalling molecules result from oxidative stress following pathogen infection (Mueller *et al*., 2008). In contrast, other pathogen‐related signalling molecules such as salicylic acid and methyl jasmonate, associated with systemic resistance and tissue injury respectively, selectively induced UGTs belonging predominately to groups D and L. UGT induction by the safener was distinct from that resulting from exposure to chemicals that inhibit plant hormone signalling; namely 2,3,5‐triiodobenzoic acid (auxin transport), silver nitrate (ethylene synthesis) and 5‐(3,4‐dichlorophenyl)furan‐2‐yl)‐piperdinlyl‐methanethiol (abscisic acid‐mediated gene induction). The protein synthesis inhibitor cycloheximide was the only chemical that caused the suppression of multiple UGTs. The gene expression data for rice in Genevestigator consisted of fewer studies representing different experimental conditions to those studied in Arabidopsis. It was therefore impossible to make direct comparisons of UGT responsiveness between the two species. Overall, the UGTs in rice shared much less uniformity in their response to the top 25 conditions with fewer family members up‐regulated than determined in Arabidopsis (Figure 4). However, as with Arabidopsis, the UGTs up‐regulated by the greatest number of conditions were UGT98B1, UGT701A1, UGT703A2 and UGT703A4, all members of group D and UGT74H3, an orthologue of the highly up‐regulated Arabidopsis UGT74E2 and a member of group L.

### Induction of other xenome genes by fenclorim in Arabidopsis and rice

**Figure 5:**
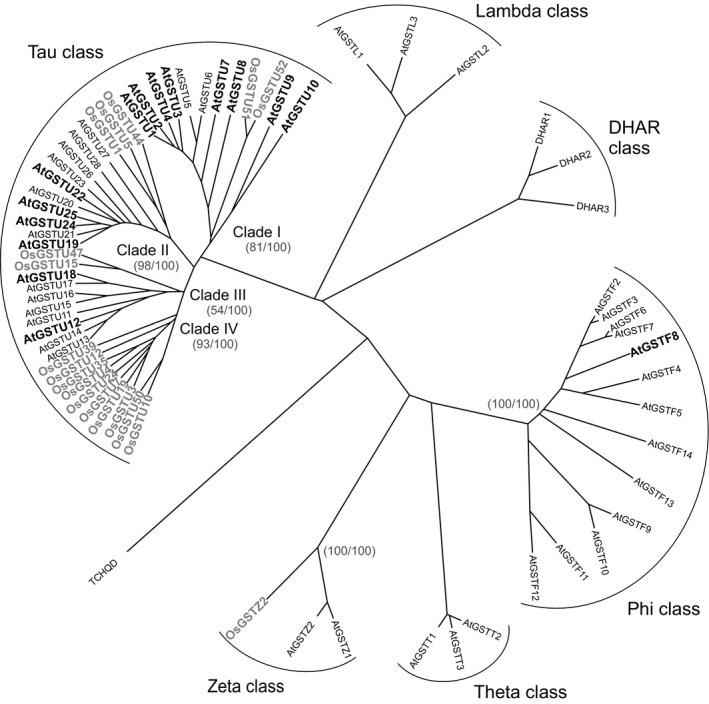
Phylogenetic tree of GSTs in cell cultures of Arabidopsis with family members induced by fenclorim shown in bold black type, with those family members induced in rice superimposed on the tree and shown in bold grey type.

**Figure 6:**
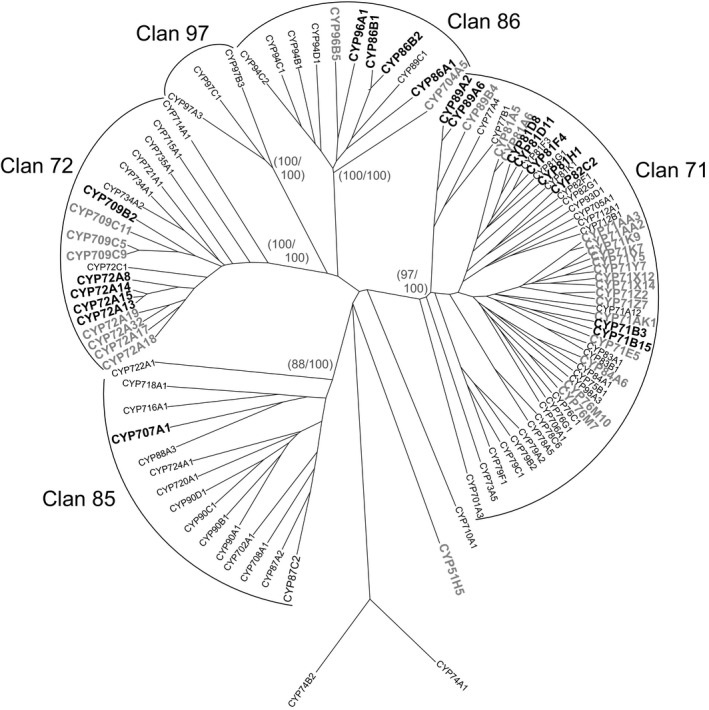
Phylogenetic tree of CYPs in cell cultures of Arabidopsis with family members induced by fenclorim shown in bold black type, with those family members induced in rice superimposed on the tree and shown in bold grey type.

To expand the analysis of safener‐inducible xenome components beyond the UGTs, a similar analysis was applied to family members of the GST (Figure 5) and CYP (Figure 6) superfamilies. In the case of the GSTs, the dominant safener‐inducible genes belonged to the tau class, with the Arabidopsis AtGSTF8 being the only phi member enhanced by fenclorim treatment in either species. This was significant, as only phi and tau class GSTs are active in detoxifying herbicides such as chloroacetanilides in higher plants (Dixon *et al*., 2009). This in turn suggested that the differential induction of tau class GSTs must underpin the enhanced tolerance to herbicides such as the chloroacetanilides determined in rice, but not in Arabidopsis. The tau class GSTs were subclassified into four clades labelled I‐IV (Figure 5). Whereas clades I to III contained both Arabidopsis and rice GSTs induced by safeners in both species, clade IV was represented by GSTs exclusively safened in rice but not in Arabidopsis (Figure 5). The most highly up‐regulated genes belonged clade I (U3 and U4 from Arabidopsis) and clade II (U24 and U25 from Arabidopsis and U1 and U5 from rice). In comparison, the rice GSTs from clade IV were much more modestly induced (<23 fold) by fenclorim. With the CYPs, safener responsiveness was determined largely in members of Clan 71 and Clan 72, with a smaller number of outliers in Clan 86. With respect to identifying major differences in the two species, in clan 71 most of the rice inducible CYPs belonged to family CYP71 whereas in Arabidopsis, the majority of up‐regulated Clan 71 CYPs were members of family CYP81. Although only two rice CYP81s were induced by fenclorim, they included CYP81A6 (up‐regulated 34‐fold) which is responsible for the metabolism of the herbicides bentazon and bensulfuron in rice (Pan *et al*., 2006). In clans 72 and 86, the evolutionary distinctness of the safener induction seen in the two species was less obvious.

## Discussion

A screen of the Arabidopsis UGT super‐family showed around 40% of its members could conjugate xenobiotics, with 13 showing activity to all three classes of acceptor groups. Of these multifunctional UGTs, three D‐group enzymes were particularly active. This may indicate that as is the case in animals (Tripathi *et al*., 2013), only a small number of UGTs are responsible for the majority of drug/xenobiotic conjugation *in planta*. In addition to the previously described OGT activity towards 2,4,5‐TCP (Brazier‐Hicks *et al*., 2007a,b), the majority of the 44 UGTs also catalysed the conjugation of 2‐chloro‐4‐trifluoromethylphenol and triclosan. UGTs active towards triclosan were of particular interest, in view of the importance of this compound as a widely biocide that has the potential to enter the environment as a pollutant. While activity towards the biocide was widespread, only one enzyme, UGT71D1, showed a selective preference towards it as an acceptor. OGT activities towards xenobiotics were largely confined to members of the major phylogenetic groupings of D, E, H and L. In comparison with substrate selectivity towards natural products, all but one of the 40 UGTs in groups D, E, H and L that glucosylated xenobiotic phenols were also active towards hydroxycoumarins (Lim *et al*., 2003). Similarly, the members of these groups that failed to conjugate hydroxycoumarins showed no activity towards 2,4,5‐TCP. When quercetin was used to screen the panel of fusion proteins, 29 UGTs were found to have activity towards at least one of the five available hydroxyl groups of the flavonol (Lim *et al*., 2004). As determined in the current study, 24 of these flavonol UGTs were also active towards 2,4,5‐TCP, with the majority residing in group D and E and a smaller number in groups H and L. The UGTs in this study that showed activity towards quercetin are labelled with an asterisk in Table 1. This demonstrates that a large subset of UGTs present in groups D, E H and L act on a diverse range of phenolic acceptors. A good example is UGT88A1, which was the only UGT to have activity towards all 11 panel substrates and the only enzyme tested that could glucosylate quercetin at four of five of the available hydroxyl positions (Lim *et al*., 2004). The loss of broad‐ranging acceptor specificities in other group members is consistent with their evolution to perform specific functions. For example, in group H a loss of function event occurred prior to the expansion and specialization of the group containing UGT76B1, UGT76Cs and UGT76F2 (Lim *et al*., 2003). Thus, UGT76B1 has a specific function in the modulation of plant defence and senescence and acts by controlling the availability of isoleucic acid and salicylic acid by glucosylation (von Saint Paul *et al*., 2011). In contrast, UGT76C1 and UGT76C2 are involved in the regulation of cytokinin homoeostasis, glucosylating at the N^7^ and N^9^ positions (Hou *et al*., 2004). Whereas a consequence of the specialization of the group H enzymes appears to be the loss of conjugating activity towards synthetic acceptors, this is not the case with other UGTs, notably those active as NGTs towards aniline substrates. The screen of the Arabidopsis UGTs demonstrated that this activity was only determined in the E group, most notably with UGT72B1. Recently, it has been reported that UGT72B1 in Arabidopsis has a very specific role in monolignol biosynthesis during cell wall lignification (Lin *et al*., 2016). A structural characterization of UGT72B1 and an OGT orthologue from *Brassica napus* showed that the ability to *N*‐glucosylate 3,4‐DCA is a consequence of a specific noncanonical arrangement of the active site of the Arabidopsis enzyme (Brazier‐Hicks *et al*., 2007a,b). Unpredictably, in this instance a UGT showing very high activities towards *N‐*acceptor xenobiotics is a consequence of an apparently random mutation in an enzyme with a specialized function in endogenous metabolism. Low levels of conjugating activity towards thiol‐bearing acceptors were determined in many of the 44 UGTs. High SGT conjugating activity was only associated with UGT74F1, UGT74B1 and UGT84B1, exceeding OGT activity in each case. Interestingly, UGT74B1 which had activity towards all three xenobiotic *S‐*acceptors is involved in glucosinolate biosynthesis, where it conjugates thiohydroximates (Grubb *et al*., 2004). Thus, unlike the xenobiotic NGT activity that has arisen due to a chance mutation of an OGT, these more specialized UGTs have evolved specifically to conjugate *S*‐acceptors.

The Genevestigator studies, using the profile of safener‐induced UGTs as a searching tool, demonstrated the conserved inducibility of members of the D and to a lesser extent L groups in their responsiveness to diverse stresses in both Arabidopsis and rice. D and L group UGTs have also been identified in unbiased screens following chemical treatments of Arabidopsis with the acid‐extractable organic fraction of oil sands process‐affected water (Widdup *et al*., 2015), the explosive trinitrotoluene (Gandia‐Herrero *et al*., 2008) and the allelochemical benzoxazolin‐2(3H)‐one (Baerson *et al*., 2005). Similarly, exposure of *Brachypodium distrachyon* and barley to the mycotoxin deoxyinvlenol (DON) from *Fusarium graminearum* resulted in the up‐regulation of group D and L transcripts (Schweigher *et al*., 2013). In Arabidopsis, the core components of the UGT‐stress‐inducible response were the D‐group enzymes UGT73B3, UGT73B4 and UGT73B5 and the L group member UGT74E2. Chemical stress treatments of Arabidopsis also identified UGT73B4 and UGT73B5, along with UGT73B2 and UGT75B1, as being up‐regulated following treatment with benzoxazolin‐2(3H)‐one and a range of xenobiotics (Baerson *et al*., 2005). The D‐group UGTs which were induced following exposure to chemicals all showed some OGT and low SGT activity towards xenobiotics, suggesting that these enzymes had a direct protective function in detoxifying foreign compounds. However, these same enzymes are also induced by pathogens, with the knockout of UGT73B3 and UGT73B5 in Arabidopsis reducing resistance to *Pseudomonas syringae* (Langlois‐Meurinne *et al*., 2005). This suggests that these UGTs are more likely to have protective roles in conjugating endogenous toxic metabolites produced as a ‘down‐stream’ consequence of both biotic and abiotic stress. Certainly, UGT74E2 which was the most highly and widely up‐regulated UGT in group L had no glycosylating activity towards the panel of xenobiotics, although it is known to *C‐* and *O‐*conjugate reactive TNT catabolites (Gandia‐Herrero *et al*., 2008). Consistent with a role in regulating the bioactivity of endogenous stress metabolites, UGT74E2 is induced by peroxide exposure and is a modulator of water stress tolerance (Tognetti *et al*., 2010). However, in contrast to members of group D, knock‐down of UGT74E2 results in enhanced resistance to *P. syringae* indicating they must act on very different groups of defence metabolites (Park *et al*., 2011).

The species‐specific gene induction of different UGTs and other xenome components by fenclorim was revealing. In the case of the fenclorim‐inducible UGTs, a similar range of transcripts has also been reported to be up‐regulated following exposure to the maize safener benoxacor (Baerson *et al*., 2005) as well as a mixture of mefenpyr‐diethyl and isoxadifen, which are used to safen wheat and maize, respectively (Behringer *et al*., 2011). This suggests that in Arabidopsis, while the quantitative response to different safener chemistries varies, the profile of genes induced is conserved. Of the 22 UGTs induced by fenclorim, nine showed no conjugating activity towards 2,4,5‐TCP and of the 13 others, those showing the greatest enhancement had only low OGT and SGT activity. This presumably accounted for the very modest increases in OGT specific activity determined in Arabidopsis plants and cultures treated with a range of safeners (Edwards *et al*., 2005). On comparing the profile of safener‐inducible UGTs in rice and Arabidopsis, the largest numbers of inducible genes resided in the D and L classes. This suggests that the respective transcriptional regulatory pathways controlling the expression of the D and L families in the two species must be conserved. In Arabidopsis, safening is known to use both salicylic acid and TGA‐transcription factor linked signalling pathways (Behringer *et al*., 2011). However, in both species distinctly evolved branches of the respective families were safener inducible. In the case of the D group, this resulted in a greater diversity of UGTs being induced in rice than in Arabidopsis. It was also potentially significant that safening in rice caused the enhanced expression of the group A UGT91C1 with its links to cytokinin metabolism. By comparing the inducibility by fenclorim of UGT, GST and CYP superfamilies in Arabidopsis and rice, it was clear that the major groupings of safened genes were evolutionarily conserved in both species. For the UGTs, safened genes largely fell within the D and L groups, for the GSTs within the tau class and for the CYPs within clans 71 and 72. Given the very different outcomes of safener treatment with respect to enhanced herbicide tolerance in responsive cereals as compared with nonresponsive broad‐leaf crops, this is a surprising observation. Firstly, it suggests that while the significance to the physiology of higher plants of safening remains obscure, the associated signalling pathways are both extensive and conserved in monocots and dicots. It also infers that it is likely that the enhanced herbicide detoxification seen following safener treatment is most likely due to a small number of up‐regulated enzymes that possess unusually high detoxifying activities towards xenobiotics. If this were not the case, it would be very unlikely to have so many xenome family members up‐regulated in both species with such different outcomes with respect to enhanced herbicide metabolism and selectivity. Evidence for this induction of ‘super‐detoxifying’ enzymes can be determined in the results obtained for the GSTs and CYPs. In the case of the GSTs, orthologues of the rice safener‐inducible clade IV enzymes in rice are known to be highly active in detoxifying the herbicides fenoxaprop and dimethenamid in wheat (Thom *et al*., 2002), as well as chloroacetanilides in maize (Rossini *et al*., 1998). With the CYPs, the orthologue of the fenclorim‐inducible rice CYP81A6 in maize termed CYP81A9 has been implicated in sensitivity to herbicides with five different modes of action in sweetcorn cultivars (Pataky *et al*., 2008). The importance of related CYPs in herbicide detoxification has been further reinforced by the observation that populations of the weeds *Echinochloa phyllopogon* and *Lolium rigidum,* that are resistant to graminicides show elevated expression of Clan 71 and 72 CYPs (Duhoux and Délye, 2013; Iwakami *et al*., 2014).

By extending these studies on gene function and regulation into other xenobiotic metabolizing enzymes, a complete picture of how plant detoxification systems function in different species is now emerging that will prove an invaluable resource in selecting plants for roles in phytoremediation of organic pollutants as well as understanding the molecular basis of herbicide selectivity in crops and weeds.

## Experimental procedures

### Plant studies

N1 rice suspension cultures were treated with 100 μm fenclorim and harvested after 4 and 24 h, with total RNA extracted from the plant tissue for microarray analysis (Skipsey *et al*., 2011). Analysis of the rice microarray data was carried out using the same software, procedures and settings as for the Arabidopsis microarray performed in Skipsey *et al*. (2011).

### Bioinformatic analysis

Phylogenetic analysis was performed by aligning protein sequences using ClustalW2 and the phylogeny inference package Phylip‐3.695 (Felsenstein, 1989). PRODIST was used to compute the distance measure using the Jones–Taylor–Thornton matrix and NEIGHBOR to construct the tree using the UPGMA (average linkage clustering) algorithm. Bootstrap analysis was performed using SEQBOOT to produce multiple data sets (100) and CONSENSE to produce a consensus tree. For Genevestigator (Hruz *et al*., 2008), the expression signature tool was imputed with UGT genes and their relative induction (experimental versus control) defined. Similarities with other treatments were then calculated based on Euclidean distance.

### Expression of GST‐UGTs

The GST‐UGT expression plasmids (Lim *et al*., 2001) were transformed into *E. coli* Rosetta (DE3) cells and grown in LB medium at 37 °C to an OD_600_ of 0.6. The cells were then induced by the addition of 0.1 mm IPTG and grown at 25 °C for 20 h. To prepare the crude cell lysate for assay, pellets from 5 mL of culture were resuspended in 1 mL of 20 mm Tris‐HCl, 2 mm DTT, pH 8.0, sonicated and centrifuged to remove cell debris. The concentration of the GST‐UGT fusion protein was determined by assaying 25 μL of the crude cell lysate for GST activity using 1‐chloro‐2,4‐dinitrobenzene (CDNB) as substrate (Edwards *et al*., 2005).

### UGT enzyme assay

Crude protein from plant extracts was prepared as described in Brazier‐Hicks *et al*. (2007a,b). UGT assays were carried out in a total volume of 75 μL comprising 20 mm Tris‐HCl, 2 mm DTT, pH 8.0 containing 66 μm xenobiotic substrate added in methanol and UDP‐[^14^C‐glucose] (50 000 dpm; specific activity 11 MBq/μmol). In the case of recombinant UGT preparations, between 2 and 10 μl of the crude bacterial lysates was added, whereas for plant extracts, 100 μg of crude protein was used. In each case, the assays were incubated at 30 °C for 20 min, prior to quenching the reaction, extracting with ethyl acetate and determining the radioactivity in the organic phase by liquid scintillation counting (Brazier‐Hicks *et al*., 2007a,b).

### Purification of GST fusion proteins

Crude lysates of induced pGEX‐5X‐1 and pGEX‐UGT72B1 were prepared as described above and applied to a GSTrap (1 mL) affinity column (GE Healthcare). The bound GST/fusion was recovered using 5 mm reduced glutathione, with the protein content determined by measuring absorbance of purified proteins at 280 nm. Quantitative Western blotting was performed using wet blotting to transfer polypeptides to Hybond ECL membrane (GE Healthcare, Little Chalfont, Bucks, UK). The blot was then probed with an anti‐GST‐antibody (GE Healthcare) coupled to horseradish peroxidase and the bound complex visualized by chemifluorescence using ECL‐Plus Western Blotting Detection Reagent (GE Healthcare) coupled to a FujiFilm FLA‐3000 Image Analyzer (Fuji Photo Film (UK) Ltd., Bedford, UK).

## Supporting information


**Figure S1** Determination of the amount of fusion protein in crude lysates of E.coli expressing GST‐UGT72B1.
**Table S1** Full list of xenobiotic compounds tested as acceptors for glucosylation using crude protein extracts from Arabidopsis.
**Table S2** Glucosyltransferase activity toward 2,4,5‐TCP and 3,4‐DCA in crude protein extracts isolated from a range of Arabidopsis tissue types.
**Table S4** Summary of GST and CYP genes in Arabidopsis root cultures and rice N1 cell cultures perturbed by treatment with fenclorim for four and 24 hours.

**Table S3** Differentially expressed genes from rice cell cultures treated with fenclorim for four and 24 hours.
