# Supplementary material for: Substrate specificity and safener inducibility of the plant UDP‐glucose‐dependent family 1 glycosyltransferase super‐family
Source: Plant Biotechnol J. 2017 Jul 17;16(1):337–48. doi: 10.1111/pbi.12775 (PMC5785338; doi:10.1111/pbi.12775)
Supplement: Supplementary file 1 — Figure S1 Determination of the amount of fusion protein in crude lysates of E.coli expressing GST‐UGT72B1. Table S1 Full list of xenobiotic compounds tested as acceptors for glucosylation using crude protein extracts from Arabidopsis. Table S2 Glucosyltransferase activity toward 2,4,5‐TCP and 3,4‐DCA in crude protein extracts isolated from a range of Arabidopsis tissue types. Table S4 Summary of GST and CYP genes in Arabidopsis root cultures and rice N1 cell cultures perturbed by treatment with fenclorim for four and 24 hours. [file PBI-16-337-s002.docx]

**Supporting information**

**Table S1.** Full list of xenobiotic compounds tested as acceptors for glucosylation by crude protein extracts from Arabidopsis. Their industrial use or status as pollutants is given in the footnote.

| Groups available for  glucosylation | Compounds tested for glucosyltransferase activity |
| --- | --- |
| -OH | Bromoxynil^h^, 2-Chlorobenozoic acid^hm^, 2-Chloro-3-trifluoromethylphenol^hma^, Ioxynil^h^, 1-Naphthol^hm^,  4-Nitrophenol^pi,fi^, Tetrabromobisphenol^fr^, 2,4,5-  Trichlorophenol^hi^, Triclosan^am^, |
|  |  |
| -NH_2_ | Chloridazon^h^, 3,4-Dichloroaniline^hi^, Metribuzin^h^, 4-Nitroaniline^pi^, Sulfadiazine^p^ |
|  |  |
| -SH | 4-Chlorothiophenol^im^, *N,N*-Dimethyldithiocarbamate^f^,  2-Napthlalenethiol^fa^ |
|  |  |
| -COOH | Chlorobenzoic acid^hm^, 3,5-Dichlorobenzoic acid^hm^, 2,2-Dichlorovinyl-2,2-dimethylcyclopropane carboxylic acid^im^, Diclofenac^p^, Ibuprofen^p^, MCPA^h^, Perfluorooctanoic acid^r^,  3-Phenoxybenzoic acid^im^, Quizalopfop^h^. |
|  |  |
| -OH, -NH_2_ | Hymexazole^f^, Picloram^h^ |
|  |  |
| -OH, -COOH | Difunisal^p^, 2-(4-Hydroxyphenoxy)propanoic acid^hi^_,_ |

^am^ anti-microbial, ^f^ fungicide, ^fa^ flavouring agent, ^fr^ flame retardant, ^h^ herbicide, ^hi^ herbicide intermediate, ^hm^ herbicide metabolite, ^hma^ herbicide metabolite analogue, ^im^ insecticide intermediate, ^p^ pharmaceutical, ^pi^ pharmaceutical intermediate, ^r^ repellent.

**Table S2.** Glucosyltransferase activity toward 2,4,5-TCP and 3,4-DCA in crude protein extracts isolated from a range of Arabidopsis tissue types.

|  | Specific activity (fkat mg^-1^ crude protein) | |
| --- | --- | --- |
|  | 2,4,5-TCP | 3,4-DCA |
| Cell suspension culture | **468** ± 58 | **952** ± 9 |
| Root cultures | **231** ± 24 | **751** ± 9 |
| Foliage | **461** ± 74 | **1092** ± 8 |
| Roots | **102** ± 12 | **169** ± 10 |
| Flowers | **392** ± 34 | **974** ± 101 |
| Siliques | **260** ± 16 | **724** ± 19 |

**Table S4.** Summary of GST and CYP genes in Arabidopsis root cultures and rice N1 cell cultures perturbed by treatment with fenclorim for four and 24 hours.

|  |  |  | Induction of xenome transcripts (n-fold) | | | | | | | |
| --- | --- | --- | --- | --- | --- | --- | --- | --- | --- | --- |
|  |  |  | Arabidopsis root culture | | | | Rice cell culture | | | |
| Xenome family | Classification | Subclassification | Gene | Locus | 4 h | 24 h | Gene | Locus | 4 h | 24 h |
| Glycosyltransferases | Group A | UGT91 | UGT91C1 | At5g49690 | 4.4 | 3.4 |  |  |  |  |
| (UGTs) | Group B | UGT89 | UGT89A2 | At5g03490 | 7.1 | 4.6 |  |  |  |  |
|  | Group D | UGT73 | UGT73B1 | At4g34138 | 8.7 | 8.3 |  |  |  |  |
|  |  |  | UGT73B2 | At4g34135 | 24.5 | 13.5 |  |  |  |  |
|  |  |  | UGT73B4 | At2g15490 | 114.1 | 51.6 |  |  |  |  |
|  |  |  | UGT73B5 | At2g15480 | 79.8 | 20.6 |  |  |  |  |
|  |  |  | UGT73C1 | At2g36750 | 3.9 | 2.5 |  |  |  |  |
|  |  |  | UGT73C7 | At3g53160 | 3.4 | 6.4 |  |  |  |  |
|  |  |  | UGT73D1 | At3g53150 | 6.3 | 3.2 |  |  |  |  |
|  |  | UGT98 |  |  |  |  | UGT98B1 | LOC_Os01g08090 | 37.5 | 3.2 |
|  |  | UGT99 |  |  |  |  | UGT99B3 | LOC_Os10g09990 | 3.9 | 1.2 |
|  |  | UGT701 |  |  |  |  | UGT701A1 | LOC_Os04g44250 | 3.8 | 2.6 |
|  |  | UGT703 |  |  |  |  | UGT703A1 | LOC_Os01g45140 | 5.2 | 3.3 |
|  |  |  |  |  |  |  | UGT703A2 | LOC_Os01g45110 | 10.7 | 2.9 |
|  |  |  |  |  |  |  | UGT703A4 | LOC_Os01g41430 | 3.6 | 0.4 |
|  | Group E | UGT71 | UGT71B6 | At3g21780 | 4.4 | 1.5 |  |  |  |  |
|  |  | UGT72 |  |  |  |  | UGT72F1 | LOC_Os05g12450 | 3.5 |  |
|  |  |  |  |  |  |  | UGT72J2 | LOC_Os01g43270 | 5.4 | 0.5 |
|  | Group G |  | UGT85A1 | At1g22400 | 6.8 | 2.5 | UGT85E2 | LOC_Os02g51910 | 6.2 | 13.0 |
|  | Group H | UGT76 | UGT76B1 | At3g11340 | 5.4 | 2.3 |  |  |  |  |
|  |  |  | UGT76E11 | At3g46670 | 5.5 | 3.2 |  |  |  |  |
|  |  |  | UGT76E12 | At3g46660 | 5.1 | 3.9 |  |  |  |  |
|  |  | UGT710 |  |  |  |  | UGT710C2 | LOC_Os07g13770 | 6.6 | 5.2 |
| Glycosyltransferases | Group J | UGT87 | UGT87A2 | At2g30140 | 11.8 | 5.1 | UGT87C1 | LOC_Os01g59110 | 12.0 | 1.5 |
| (UGTs) continued | Group K | UGT86 | UGT86A1 | At2g36970 | 6.3 | 5.3 |  |  |  |  |
|  | Group L | UGT74 | UGT74E2 | At1g05680 | 138.6 | 42.9 | UGT74H3 | LOC_Os09g34250 | 4.2 |  |
|  |  |  | UGT74F2 | At2g43820 | 4.1 | 2.8 | UGT74H4 | LOC_Os09g34230 | 5.6 | 1.4 |
|  |  | UGT75 | UGT75B1 | At1g05560 | 27.3 | 13.4 | UGT75E1 | LOC_Os11g04860 | 4.4 | 2.2 |
|  |  |  | UGT75D1 | At4g15550 | 6.3 | 4.4 | UGT75K1 | LOC_Os01g08440 | 16.5 | 4.5 |
|  |  | UGT84 | UGT84A2 | At3g21560 | 3.7 | 2.2 | UGT84C1 | LOC_Os02g09510 | 5.7 |  |
|  |  |  | UGT84A3 | At4g15490 | 5.9 | 6.0 | UGT84D1 | LOC_Os05g47950 | 3.1 |  |
|  | Group O | UGT93 |  |  |  |  | UGT93B* | LOC_Os04g46970 | 4.5 |  |
| Glutathione transferases | Phi class |  | AtGSTF8 | At2g47730 | 3.8 | 2.6 |  |  |  |  |
| (GSTs) | Tau class | Clade I | AtGSTU1 | At2g29490 | 14.4 | 14.4 | OsGSTU50 | LOC_Os10g38740 | 9.3 | 1.3 |
|  |  |  | AtGSTU2 | At2g29480 | 24.5 | 9.1 | OsGSTU51 | LOC_Os11g03210 | 4.3 | 11 |
|  |  |  | AtGSTU3 | At2g29470 | 441.6 | 32.4 |  |  |  |  |
|  |  |  | AtGSTU4 | At2g29460 | 193.0 | 5.6 |  |  |  |  |
|  |  |  | AtGSTU7 | At2g29420 | 8.6 | 5.1 |  |  |  |  |
|  |  |  | AtGSTU8 | At3g09270 | 11.6 | 9.7 |  |  |  |  |
|  |  |  | ATGSTU9 | At5g62480 | 3.9 | 1.4 |  |  |  |  |
|  |  |  | AtGSTU10 | At1g74590 | 7.1 | 2.3 |  |  |  |  |
|  |  | Clade II | AtGSTU19 | At1g78380 | 4.1 | 3.5 | OsGSTU1 | LOC_Os03g57200 | 449.8 | 8.7 |
|  |  |  | AtGSTU22 | At1g78340 | 10.0 | 3.4 | OsGSTU5 | LOC_Os09g20220 | 274.7 | 193.2 |
|  |  |  | AtGSTU24 | At1g17170 | 388.1 | 98.5 | OsGSTU44 | LOC_Os03g44170 | 11.4 | 3.4 |
|  |  |  | AtGSTU25 | At1g17180 | 254.3 | 14.8 |  |  |  |  |
|  |  | Clade III | AtGSTU11 | At1g69930 | 13.8 | 6.5 | OsGSTU15 | LOC_Os10g38140 | 17.9 | 10.2 |
|  |  |  | AtGSTU12 | At1g69920 | 40.6 | 2.1 | OsGSTU47 | LOC_Os10g34020 | 18.0 | 3.1 |
|  |  |  | AtGSTU18 | At2g02390 | 4.4 | 2.8 |  |  |  |  |
|  |  | Clade IV |  |  |  |  | OsGSTU3 | LOC_Os10g38501 | 4.5 | 3.3 |
|  |  |  |  |  |  |  | OsGSTU4 | LOC_Os10g38495 | 22.9 | 6.5 |
| Glutathione transferases | Tau class  continued | Clade IV continued |  |  |  |  | OsGSTU7 | LOC_Os10g38580 | 3.7 | 0.7 |
| (GSTs) continued |  |  |  |  |  |  | OsGSTU10 | LOC_Os10g38780 | 10.3 | 3.5 |
|  |  |  |  |  |  |  | OsGSTU12 | LOC_Os10g38360 | 2.3 | 1.6 |
|  |  |  |  |  |  |  | OsGSTU19 | LOC_Os10g38340 | 12 | 3.3 |
|  |  |  |  |  |  |  | OsGSTU24 | LOC_Os10g38470 | 5.2 | 4.0 |
|  |  |  |  |  |  |  | OsGSTU35 | LOC_Os01g72130 | 6.3 | 1.4 |
|  |  |  |  |  |  |  | OsGSTU39 | LOC_Os01g49720 | 13.9 | 1.2 |
|  |  |  |  |  |  |  | OsGSTU52 | LOC_Os12g02960 | 5.9 | 1.2 |
|  | Zeta class |  |  |  |  |  | OsGSTZ2 | LOC_Os12g10730 | 15.1 | 1.9 |
| Cytochrome P450s  (CYPs) | Clan 51 |  |  |  |  |  | CYP51H5 | LOC_Os02g02230 | 4.6 | 0.5 |
|  | Clan 71 | CYP71 | CYP71B3 | At3g26220 | 3.1 | 1.9 | CYP71AA2 | LOC_Os01g72760 | 3.4 | 1.6 |
|  |  |  | CYP71B15 | At3g26830 | 30.2 | 1.1 | CYP71AA3 | LOC_Os01g72740 | 3.7 | 0.7 |
|  |  |  |  |  |  |  | CYP71AK1 | LOC_Os09g36070 | 5.8 | 0.6 |
|  |  |  |  |  |  |  | CYP71E5 | LOC_Os12g32850 | 4.1 | 1.8 |
|  |  |  |  |  |  |  | CYP71K7 | LOC_Os06g43480 | 4.0 | 1.8 |
|  |  |  |  |  |  |  | CYP71K9 | LOC_Os06g43430 | 3.9 | 0.7 |
|  |  |  |  |  |  |  | CYP71X12 | LOC_Os02g09190 | 6.3 | 0.9 |
|  |  |  |  |  |  |  | CYP71X14 | LOC_Os02g38940 | 3.6 | 0.8 |
|  |  |  |  |  |  |  | CYP71Y5 | LOC_Os06g43350 | 4.1 | 2.2 |
|  |  |  |  |  |  |  | CYP71Y7 | LOC_Os06g43304 | 3.2 | 1.2 |
|  |  |  |  |  |  |  | CYP71Z2 | LOC_Os07g11739 | 3.3 | 0.8 |
|  |  |  |  |  |  |  | CYP71Z7 | LOC_Os02g36190 | 3.3 | 0.4 |
|  |  | CYP76 |  |  |  |  | CYP76M7 | LOC_Os09g35940 | 3.6 | 2.7 |
|  |  |  |  |  |  |  | CYP76M10 | LOC_Os02g36110 | 7.3 | 0.2 |
|  |  | CYP78 |  |  |  |  | CYP78C6 | LOC_Os09g35940 | 4.3 | 0.2 |
|  |  | CYP81 | CYP81D8 | At4g37370 | 41.2 | 11.3 | CYP81A5 | LOC_Os03g55230 | 9.7 | 2.4 |
|  |  |  | CYP81D11 | At3g28740 | 106.7 | 125.5 | CYP81A6 | LOC_Os03g55240 | 33.7 | 3.1 |
| Cytochrome P450s  (CYPs) continued | Clan 71 | CYP81  continued | CYP81F2 | At5g57220 | 17.7 | 1.6 |  |  |  |  |
|  | continued |  | CYP81F4 | At4g37410 | 7.4 | 10.1 |  |  |  |  |
|  |  |  | CYP81G1 | At5g67310 | 5.0 | 3.4 |  |  |  |  |
|  |  |  | CYP81H1 | At4g37310 | 3.6 | 3.6 |  |  |  |  |
|  |  | CYP82 | CYP82C2 | At4g31970 | 183.0 | 21.6 |  |  |  |  |
|  |  | CYP84 |  |  |  |  | CYP84A6 | LOC_Os03g02180 | 4.6 | 1.0 |
|  |  | CYP89 | CYP89A2 | At1g64900 | 21.3 | 10.4 | CYP89B4 | LOC_Os10g37100 | 9.7 | 1.4 |
|  |  |  | CYP89A6 | At1g64940 | 18.3 | 19.2 |  |  |  |  |
|  | Clan 72 | CYP72 | CYP72A8 | At3g14620 | 18.7 | 3.0 | CYP72A17 | LOC_Os01g43700 | 4.0 | 1.4 |
|  |  |  | CYP72A13 | At3g14660 | 7.1 | 4.4 | CYP72A18 | LOC_Os01g43710 | 15.2 | 1.2 |
|  |  |  | CYP72A14 | At3g14680 | 3.6 | 1.7 | CYP72A19 | LOC_Os01g43720 | 4.4 | 1.5 |
|  |  |  | CYP72A15 | At3g14690 | 9.9 | 5.8 | CYP72A32 | LOC_Os01g41810 | 3.7 | 1.2 |
|  |  | CYP709 | CYP709B2 | At2g46950 | 21.8 | 14 | CYP709C5 | LOC_Os07g44140 | 51.2 | 14.2 |
|  |  |  |  |  |  |  | CYP709C9 | LOC_Os07g23570 | 78.9 | 4.3 |
|  |  |  |  |  |  |  | CYP709C11 | LOC_Os06g09220 | 5.4 | 0.9 |
|  |  | CYP734 |  |  |  |  | CYP734A2 | LOC_Os02g11020 | 5.3 | 0.5 |
|  | Clan 85 | CYP87 |  |  |  |  | CYP87C2 | LOC_Os03g45619 | 6.7 | 2.6 |
|  |  | CYP707 | CYP707A1 | At4g19230 | 3.3 | 1.9 |  |  |  |  |
|  | Clan 86 | CYP86 | CYP86A1 | At5g58860 | 3.7 | 12.0 |  |  |  |  |
|  |  |  | CYP86B1 | At5g23190 | 6.7 | 7.8 |  |  |  |  |
|  |  |  | CYP86B2 | At5g08250 | 7.2 | 3.7 |  |  |  |  |
|  |  | CYP94 |  |  |  |  | CYP94C2 | LOC_Os11g05380 | 3.6 | 1.5 |
|  |  | CYP96 |  |  |  |  | CYP96B5 | LOC_Os03g04660 | 3.6 | 1.3 |
|  |  | CYP704 |  |  |  |  | CYP704A5 | LOC_Os10g38110 | 3.2 | 1.7 |
|  | Clan 710 | CYP710 | CYP710A1 | At2g34500 | 23.5 | 7.5 |  |  |  |  |

**Figure S1.** Determination of the amount of fusion protein in crude lysates from *E.coli* expressing GST-UGT72B1 using GST activity. A. The linear relationship between the volume of lysate loaded onto a PAGE gel and the amount of fusion present as determined using an anti-GST-serum linked to an ECL plus immunoblot assay. B. The relationship between the chemifluorescent determined by western blotting with the GST enzyme activity associated with the respective sample.
